# Chemical Pleurodesis in the Treatment of Recurrent Chylothorax Due to Renal Cell Carcinoma

**DOI:** 10.7759/cureus.55363

**Published:** 2024-03-01

**Authors:** Hannah Z Weiss, Brandon W Knopp, Jeniel Parmar

**Affiliations:** 1 Division of Emergency Medicine, Florida Atlantic University Charles E. Schmidt College of Medicine, Boca Raton, USA

**Keywords:** pleural effusion, chylothorax, renal cell carcinoma, thoracentesis, chemical pleurodesis

## Abstract

Chylothorax is defined as a pleural effusion with triglyceride levels greater than 110 mg/dL and/or chylomicrons present in the pleural fluid. A chylothorax may be classified as traumatic or nontraumatic, with malignancy being the most common cause of atraumatic chylothoraces. Herein, we present the case of a 63-year-old woman with a past medical history of a mediastinal teratoma and stage III colon adenocarcinoma who presented to the emergency room with new-onset shortness of breath. A week prior to presentation, she was diagnosed with metastatic renal cell carcinoma after a retrocrural lymph node was biopsied. In the emergency department, a chest X-ray revealed a large right-sided pleural effusion, which was later diagnosed as a chylothorax based on pleural fluid analysis. Thoracentesis was performed and the patient was sent home. Three days later, the patient returned after experiencing palpitations and shortness of breath. The patient was diagnosed with recurrent chylothorax after a repeat chest X-ray and thoracentesis. The patient was ultimately treated with chemical pleurodesis. To the best of our knowledge, this case is the only reported chylothorax due to renal cell carcinoma metastasis reported in the literature. It describes the presentation and subsequent successful treatment of this rare condition with chemical pleurodesis.

## Introduction

A chylothorax is defined as a pleural effusion with triglyceride levels greater than 110 milligram/deciliter (mg/dL) and a cholesterol level less than 200 mg/dL present in the pleural fluid [1]. Chylothoraces are due to the accumulation of lymphatic fluid in the pleural space and account for 3% of all pleural effusions [2]. A chylothorax is often preceded by trauma, usually due to iatrogenic injury of the thoracic duct. Nontraumatic chylothoraces are typically due to malignancy, with lymphomas making up 61% of malignant chylothoraces [3]. Solid tumors are less likely to cause chylothorax but have been reported [4]. In order for a malignancy to lead to the development of a chylothorax, there must be an obstruction or invasion of the thoracic duct. The thoracic duct originates at the L2 level from the cisterna chyli and extends to the base of the neck. Thus, cancers that commonly originate or metastasize to that region are most likely to result in chylothorax formation. As chylothorax formation can be the first indication of malignancy, it is important to highlight its association with different malignancies including solid tumors. Additionally, a chylothorax might indicate metastasis of a previously known malignancy.

Only two cases of a chylothorax associated with renal cell carcinoma have been described in the literature. One case attributed the chylothorax formation to pazopanib use [5]. In the other case, a patient developed a chylothorax due to compression of the thoracic duct by the renal cell carcinoma mass [6]. There have been no reported cases of a chylothorax formation due to renal cell carcinoma metastasis prior to the following case. Herein, we present the case of a 63-year-old woman with a recurrent chylothorax after the recent discovery of a metastatic retrocrural lymph node with renal cell carcinoma pathology.

## Case presentation

**Figure 1:**
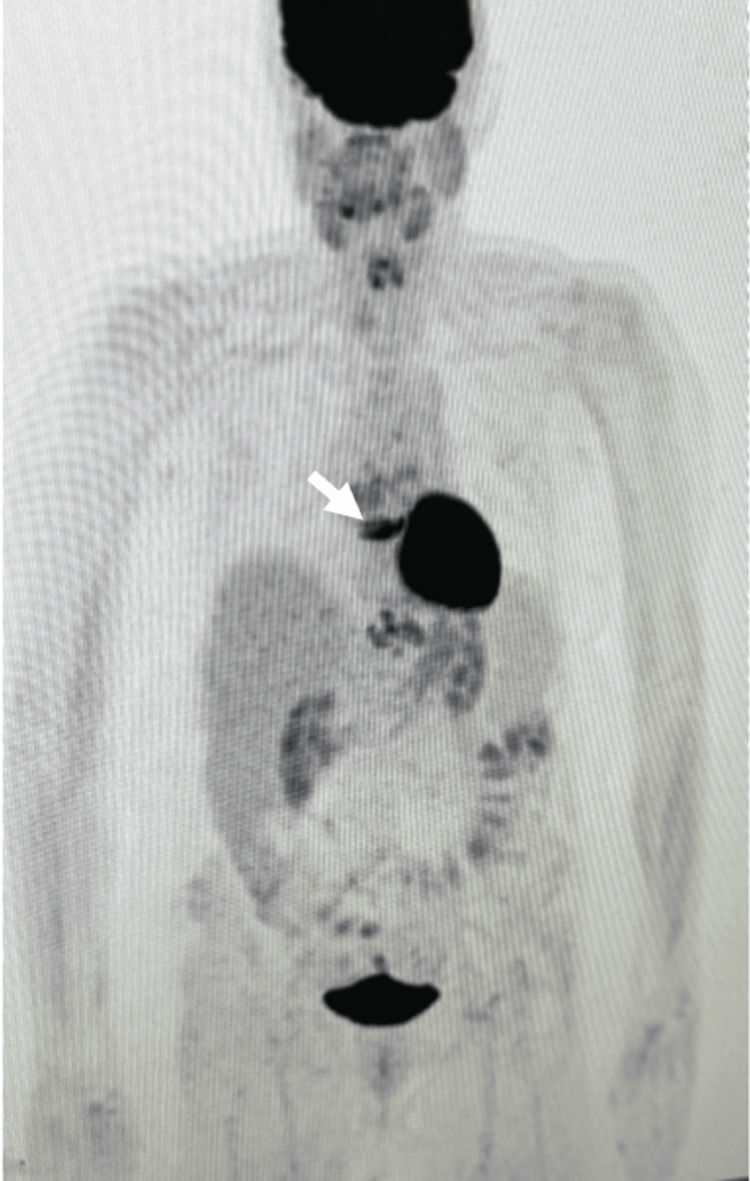
PET Scan Illustrating 1 cm Retrocrural Lymph Node PET: Positon emission tomography The white arrow points to the 1 cm retrocrural lymph node.

A 63-year-old woman with a past medical history of a mediastinal teratoma and stage III colon adenocarcinoma presented to the emergency room with new-onset shortness of breath. The mediastinal teratoma was treated by sternotomy when the patient was in her twenties, and the stage III colon adenocarcinoma was treated with partial colon resection, chemotherapy, and radiation. At the time of presentation, she was in remission from colon adenocarcinoma for over two years. The patient had a history of smoking tobacco with unknown pack years and a history of hypertension. The patient was diagnosed with new metastatic lesions of the left iliac bone, T-12 spine segment, and L-2 spine segment two months prior to presentation, but the lesion biopsy was nondiagnostic. As the original lesion biopsies were inconclusive, the patient was started on a new course of pembrolizumab and radiation, the treatment she was originally placed on for colon adenocarcinoma. A positron emission tomography (PET) scan was done that revealed a new 1 cm retrocrural lymph node (Figure 1). A week prior to the initial emergency department presentation, the patient underwent an endoscopic ultrasound with a biopsy of the lymph node. The lymph node pathology showed poorly differentiated adenocarcinoma with sarcomatoid features and extensive necrosis compatible with renal origin.

A chest X-ray taken upon emergency department presentation revealed a large right pleural effusion that was subsequently drained with thoracentesis. About 1.5 L of milky-white fluid was removed during the procedure. Fluid cytology was reported with a protein ratio of >1 and an LDH ratio of >.5, suggesting an exudate. The pleural fluid also had 2,496 mg/dL of triglycerides (Table 1). The patient was asymptomatic following thoracentesis, and the patient was discharged home.

**Table 1:** Pleural Fluid Analysis on Initial Presentation LDH: Lactate dehydrogenase; WBC: white blood cell; RBC: red blood cell

Trait	Value
Appearance	Milky
Protein (g/dL)	4
LDH (U/dL)	82
Triglycerides (mg/dL)	2,496
Cholesterol (mg/dL)	97
WBCs per microliter	466
RBCs per microlliter	3,000
Glucose (mg/dL)	197

**Figure 2:**
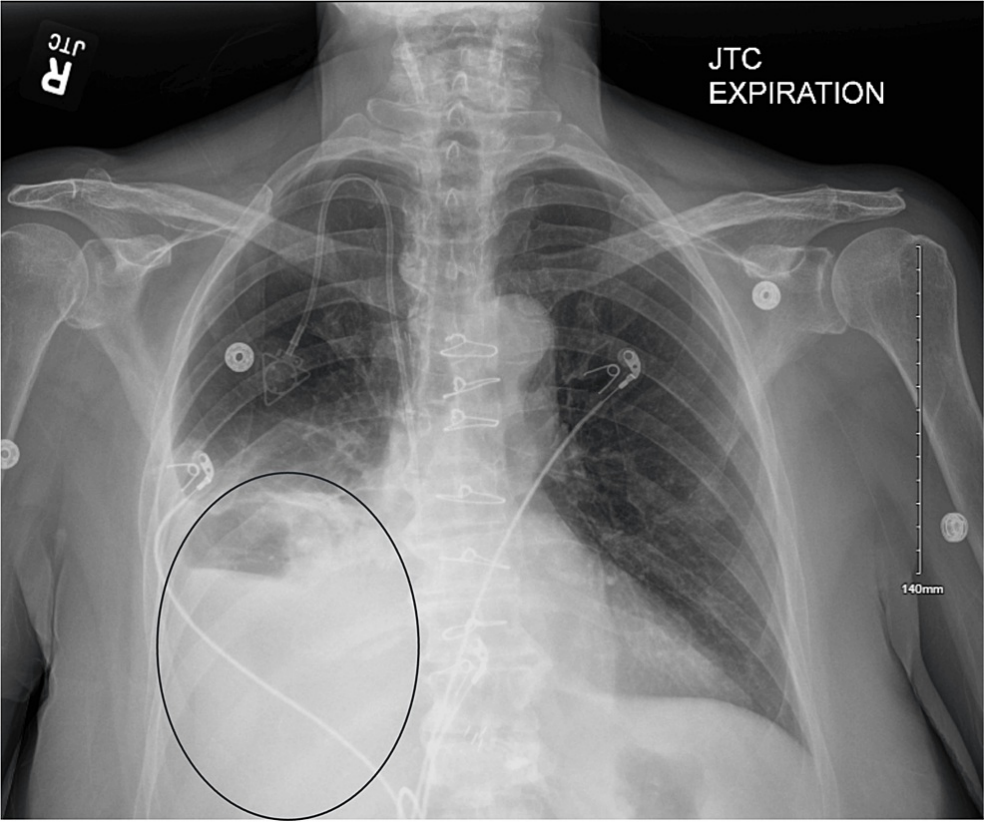
Chest X-Ray Displaying Right-Sided Pleural Effusion The encircled area demonstrates opacification in the right middle and lower lung lobes. There is also blunting of the right costophrenic angle.

Three days later, the patient returned to the emergency room after experiencing palpitations and increasing shortness of breath with ambulation for the past two days. Upon presentation, the patient was afebrile with vitals within normal limits. A physical examination revealed diminished lung sounds with rales on the right and dullness to percussion at the right lung base. A chest X-ray demonstrated a reaccumulation of the right pleural effusion (Figure 2). A right chest tube was placed with over 2 L output over the first 24 hours. The pleural fluid sample had 2,266 mg/dL triglycerides (Table 2). The patient had minimal, constant output for the next several days following chest tube placement. Four days after chest tube placement, the patient underwent bedside povidone-iodine pleurodesis and was started on a low-fat diet with medium-chain triglycerides. The chest tube was removed nine days after initial placement, and the patient was subsequently discharged.

**Table 2:** Pleural Fluid Analysis of Recurrent Chylothorax LDH: Lactate dehydrogenase; WBC: white blood cell; RBC: red blood cell

Trait	Value
Appearance	Turbid, pink
Protein (g/dL)	4
LDH (U/dL)	224
Triglycerides (mg/dL)	2,266
Cholesterol (mg/dL)	84
WBCs per microliter	1,033
RBCs per microliter	23,000
Glucose (mg/dL)	184

## Discussion

It is imperative to identify and treat chylothorax urgently as it is associated with high morbidity and mortality. Chylothorax is difficult to diagnose due to the absence of fever and pleuritic pain associated with most pleural effusions. Small chylothoraces are often asymptomatic and detected incidentally. Although our patient’s pleural fluid had the classic milky-white appearance of a chylothorax, this alone is not diagnostic as it is only seen in about half of all chylothoraces [1]. The diagnosis of chylothorax is made with a pleural fluid triglyceride content above 110 mg/dL and a cholesterol level under 200 mg/dL [1]. The pleural fluid triglyceride content in our patient was 2,496 mg/dL on the first presentation and 2,266 mg/dL on reoccurrence. Additionally, the cholesterol level was 97 mg/dL on the first presentation and 84 mg/dL on the second presentation. These values are diagnostic of a chylothorax.

The first-line treatment for chylothorax involves conservative measures, such as thoracentesis and the low-fat diet with medium-chain triglycerides utilized in this patient’s treatment plan. A low-fat diet aims to reduce the production of chyle and the subsequent leakage of triglycerides into the pleural space. Our patient’s chylothorax returned despite conservative treatments and required chemical pleurodesis. Chemical pleurodesis involves using chemical agents to fuse the visceral and parietal pleura, eviscerating the pleural space. The chemical agent triggers intrapleural inflammation, which leads to scarring. It is this scarring process that ultimately fuses the visceral and parietal pleura.

Chemical pleurodesis has been shown to successfully treat pleural effusions, most notably malignant pleural effusions [7]. To our knowledge, this is the first case of a chylothorax due to renal cell carcinoma being successfully treated with povidone-iodine pleurodesis. Povidone-iodine was chosen as the chemical agent due to its safety and efficacy [8]. It is important to note that chemical pleurodesis is only a treatment for this patient’s chylothorax. This patient later underwent radiation combined with pembrolizumab therapy to treat the renal cell carcinoma originally responsible for the chylothorax.

## Conclusions

Nontraumatic chylothorax does not always indicate a malignancy; however, it is important to consider solid and nonsolid malignancy as a potential etiology. Though lymphoma is the most common etiology, any malignancy may lead to chylothorax formation if the thoracic duct is compressed or invaded by the cancer. The chylothorax seen in this patient was attributed to the enlarged lymph node with renal cell carcinoma pathology due to its anatomic proximity to the thoracic duct. A chylothorax may be a poor prognostic factor indicating cancer progression if the chylothorax was the result of metastasis. Chemical pleurodesis is a promising treatment for refractory malignant chylothorax, though treatment for the causative malignancy is the primary treatment. This report aims to describe a presentation of a recurrent malignant chylothorax due to renal cell carcinoma metastasis and increase clinical recognition of the association between chylothorax development and the metastasis of more uncommon malignancies.
